# High Intensity-Rehabilitation in Skilled Nursing Facilities: From Theory to Practice

**DOI:** 10.1093/geroni/igaf122.2448

**Published:** 2025-12-31

**Authors:** Lauren Hinrichs-Kinney, Mattie Pontiff, Elizabeth Staton, Katie Butera, Emma Beisheim-Ryan, Dawn Magnusson, Jennifer Stevens-Lapsley

**Affiliations:** VA Eastern Colorado Health Care System, Aurora, Colorado, United States; VA Eastern Colorado Health Care System, Aurora, Colorado, United States; University of Colorado Anschutz Medical Campus, Aurora, Colorado, United States; University of Delaware, Newark, Delaware, United States; Naval Medical Center, San Diego, California, United States; University of Colorado Anschutz Medical Campus, Aurora, Colorado, United States; University of Colorado Anschutz Medical Campus, Aurora, Colorado, United States

## Abstract

Implementing evidence-based, physiologic dosing of rehabilitation in skilled nursing facilities (SNFs) is essential to improve functional outcomes in a vulnerable, medically-complex population. We evaluated an implementation program to promote high-intensity resistance rehabilitation (HIR) in SNFs.

**Methods:**

Using a prospective convergent mixed-methods design, this study 1) measured proximal (clinician knowledge, self-efficacy, and HIR perspective) and distal (HIR adoption and implementation) outcomes; 2) explored how the program influenced distal outcomes (program processes); and 3) investigated clinician factors influencing HIR implementation. Thirty-eight rehabilitation clinicians and 16 leaders in eight rural Department of Veterans Affairs SNFs participated in interviews and focus groups. Outcomes included validated questionnaires on HIR perspective (Perceived Characteristics of Intervention Scale) and adoption (Commitment to Change Scale), and study-specific questionnaires on clinician HIR knowledge, self-efficacy, and implementation.

**Results:**

The program improved clinician HIR knowledge, self-efficacy, and perspective and led to acceptable adoption rates; however, implementation was marginally affected. Only clinician HIR perspective correlated with adoption (rho = 0.47, p = 0.004). The program supported distal outcomes by keeping HIR at the forefront of clinicians’ mind, fostering positive outcome expectations, and enhancing team cohesion and accountability. Clinician creativity, adaptability, resilience, professional discipline, and previous experience influenced implementation.

**Conclusion:**

The program influenced HIR adoption primarily by enhancing clinicians’ positive perspectives of HIR. Future efforts could strengthen implementation by fostering team cohesion, accountability, and clinician creativity while assessing environmental factors influencing implementation. Strategies that enhance clinician perspective and creativity, maintain HIR top of mind, and foster team cohesion and accountability may improve adoption.

